# 

**Published:** 1872-06

**Authors:** 


### BOOK NOTICES.

"Episcopal Hymnal," published by Hurd and Houghton, New York, 491 pp. 520 hymns, besides doxologies and copious index, in large clear type, is an immense advance on any previous collections for the Episcopal Church. It is quite large enough, while it has a variety which will meet all the requirements of family, social, and public worship. In this connection we notice for the third time that admirable collection of hymns, songs, and lyrics, three hundred in number, made by the joint labors of the Rev. Dr. Deems and of that loving, lovable, and loved one who has now passed away,

PHŒBE CARY.

She was so gentle, genial, and unobtrusive! That well-remembered smile has become angelic now, and the earthly strains are made immortal. Taking it all in all, if it contained the music also, the tunes, it could with advantage be made, as its title-page truthfully indicates, a book of "Hymns for all Christians." There are not too many to be "learned by heart," as the school children express it, and if each hymn had its own familiar tune, it would not be long before "congregational singing" would be the rule, instead, as now, too often the exception; and then, too, whenever Christians met together, in any nation, on land or sea, such a voice of one accord would rise from every place of worship as would almost make it a heaven here below, for there is scarcely anything more enrapturing than a whole assembly uniting fervently, heartily, and with a will in singing to the praise of God.

"Old Schoolfellows, and What Became of Them," is a beautiful volume of the American Tract Society, 288 pp. 12mo. Illustrated, 80 cents. This attractive book, with its eleven chapters, will strike a chord in every thoughtful bosom with deeper and stronger vibrations as the years roll by. Who at threescore would not read with intense interest the lives of those who were in the "same class" with him at the country school-house, the academy, the college, the university!

"Biblical Repertory and Princeton Review," being an index volume in three parts. The first part is a history of the "Review." The second is an index to the writers of various articles, with a short biographical notice of the living, and of those who have ceased from their labors. This part will be read with exceeding interest by those who still remain, and who knew them in the flesh. Then there is an Analytical Index, and an Index to Topics. Those who wish to complete their sets of the "Review" can now do so, and learn the rates. Published by Peter Walker, 1334 Chestnut Street, Philadelphia, Penn. This "Index Volume" is of great value, especially to Presbyterian clergymen and cultivated laymen. One is surprised, in looking over the index, to see the vast stores of knowledge, of criticism and discussion contained in the old "Princeton Review," with its precious memories.

"Acoustics, Warming, and Ventilation," by ALEXANDER SWAELZER, published by Van Nostrand, 23 Murray Street, New York, 60 cents, is a valuable contribution to the discussion of subjects in which all are interested. It can be read with profit by every man who owns a house, by every man who contemplates building a house, as well as by all who are interested in the construction of public buildings, especially churches.

Lee & Shepard, New York and Boston, have issued in attractive style, "An American Girl Abroad," by ADELINE TRAFTON, illustrated by Miss L. B. Humphrey; $1.50. Also OLIVER OPTIC'S "Young America Abroad in Northern Lands, Russia and Prussia." $1.75.

"The Rival Collection of Prose and Poetry, for the use of Schools, Colleges, and Public Readers," by MARK LARKIN, published by J. W. Schermerhorn & Co., 14 Bond Street, New York, 504 pp. 8vo, $2, containing about six hundred selections in prose and poetry from the best authors. It is a good book for summer travel, as well as for the purposes designated.

"Two Family Mothers," by MARIE SOPHIE SCHWARTZ, translated from the

Swedish by Selme Borjard Marie A. Brown. Published by Lee & Shepard, Boston and New York. $1.00 paper, $1.50 in muslin.

"Pulmonary Consumption: its Nature, Varieties, and Treatment, with an Analysis of One Thousand Cases to exemplify its Duration," by C. J. B. WILLIAMS, M. D., F. R. S., Senior Consulting Physician to the Hospital for Consumption at Brompton, London; republished by Henry C. Lea, Philadelphia, Penn., for the first time in this country; it first appeared within a year in London. Its title alone will commend it to educated medical men throughout the country, and will be received by them with the confidence which it merits from the evident reliability of its statements, and the clearness with which the views of the distinguished author are expressed. The "cases" given, with their treatment, their success and failure, will be read with great interest. 8vo, 315 pp. with copious index.

"Search and Manifestation," by Lee & Shepard, Boston, 12mo, 446 pp., by the author of "Credo," L. T. TOWNSEND, D. D., Professor in the School of Theology of Boston University, dedicated to the professors and students of the institution. It is a work of great research, and will be read with deep interest by the theologians of the country, as well as by the educated laymen of the different classes of Christian men and women of orthodox predilections. $1.50.

We are constrained to notice again "Fireside Science," 283 pp. 12mo, $1.50, Hurd and Houghton, publishers Dr. NICHOLS, its author, has given the public a book which not only interests, but instructs in the useful, practical things of daily life. The very numerous extracts which have been made from it by the best class of newspapers and magazines, is a proof of the worth of the volume for the family and the fireside. Among the twenty-three subjects treated are, Origin of Springs, Rebreathed Air, Human Hair, Clothing, Skin, Bathing, Air Furnaces, Food of Plants, etc.

"The London Lancet," $5 a year, merits its former repute. Agent, WM. C. HERALD, 52 John Street, New York.

"The Boston Medical and Surgical Journal," $4, has acquired a new life and increased usefulness in the profession through the ability of its editors, Dr. F. H. Brown, and Dr. F. W. Draper, his assistant editor.

We take pleasure in commending to the profession and to families of culture, "The Boston Journal of Chemistry," monthly, only one dollar a year, single numbers, ten cents. In the April issue not less than thirty-five subjects are succinctly treated. Among these are, In-door Life, Health Hints, Culinary Recipes, — which means how to cook well. The editor uses a great many dictionary words, but for all that, this number says many plain things about familiar subjects, as, Bad Water, Bad Air, Sleeplessness, Overloading Children's Minds, etc., etc. But, — yes, here it comes, there is a "but" to everything and everybody, — how comes it that the Doctor puts the church on a level with the theatre as a cause of consumption, a cause so frequent as to make them to be singled out as the chief? The bad air of churches and theatres, he says, lays the foundation for consumption and other diseases of the throat and lungs. We don't believe a word of it. Don't believe anybody ever got consumption or any other disease by going to church; if people went to church more they wouldn't be as sick as they are. It is staying at home too much, lolling on sofas, lounging on beds, late hours, neglect of the laws of nature, and violating their first principles: these are the things which plant the seeds of consumption, in our daughters particularly, before they are twenty years old. Does nobody die of consumption but people who go to church? Why not leave the church out? What's the use of giving anybody an excuse for not going to church? People have too many excuses already. We know a young lady, high born, rich, talented, who seemed to be going into a decline. She began to go to church, went more, went oftener, got converted. We saw her put down under the water on a cold winter's day. She began to get better, and has been growing better and stronger ever since in going about doing good, hunting up Sunday-school children, visiting sick and poor people, getting up charitable concerts, receptions, lectures, readings, etc. It would seem from this that going to church, and taking an interest in church matters, and becoming a worker in promoting church objects, is a cure for consumption. Let anybody try it.

The last annual address before the Cleveland Homœopathic Hospital College was delivered February 14, 1872, by J. D. Buck, M. D., Professor of Psychology, strongly advocating the employment of educated physicians, and of a higher grade of study preparatory to attendance on medical lectures.

The Doctor seems to have in his mind's eye the ruling idea that homœopathy is synonymous with

NEW-BORN TRUTH, as contradistinguished from allopathy, which is

GRAY-HAIRED FALSEHOOD, and he looks forward hopefully to the good time coming when his favorite system of medical practice shall supersede all others; that

> "In the cycle of ages truth comes up, uppermost at last,
> And the saviors of the present are the martyrs of the past."

The address contains many valuable suggestions to medical graduates which could be profitably read by practitioners of all schools.

"The Galaxy," "Harper's Magazine," "Harper's Bazar," "Harper's Illustrated Weekly," with its circulation of a hundred and thirty thousand copies a week, "Atlantic Monthly," each four dollars a year, are always welcome.

"Baldwin's Monthly" comes regularly to hand, with its mechanical and artistic "get up" well fitting its reading matter, always sparkling and bright, and fresh and new, as "The Evening Mail" says, "with a sprinkling of poems and pungencies, all in good taste and readable." The stately "Independent" adds, "It is incomparably the best paper devoted to a specialty we have ever seen." We dropped in one day not long ago, and found that the great clothier had just treated himself to a beautiful copy of

TAINE'S ENGLISH LITERATURE, which is found on the tables of so many professional and cultivated minds.

